# Experimental infection of *Phlebotomus perniciosus* by bioluminescent *Leishmania infantum* using murine model and artificial feeder

**DOI:** 10.1590/0074-02760160100

**Published:** 2016-07-18

**Authors:** Arnaud Cannet, Mohammad Akhoundi, Gregory Michel, Pierre Marty, Pascal Delaunay

**Affiliations:** 1 Université de Nice-Sophia Antipolis, Centre Méditerranéen de Médecine Moléculaire, Nice, France; 2 Centre Hospitalier Universitaire de Nice, Hôpital de l’Archet, Service de Parasitologie-Mycologie, Nice, France

**Keywords:** experimental infection, bioluminescence, Leishmania infantum, Phlebotomus perniciosus

## Abstract

Leishmaniasis is a vector-borne disease that is transmitted by sandflies and caused by obligate intracellular protozoa of the genus *Leishmania*. In the present study, we carried out a screening on the experimental infection of *Phlebotomus pernioucus* by bioluminescent *Leishmania infantum* using murine model and artificial feeder. We developed a real-time polymerase chain reaction (RT-PCR)-based method to determine individually the number of *Leishmania* promastigotes fed by infected flies. Among 1840 new emerged female sand flies, 428 were fed on the infected mice. After their death, they were analysed individually by RT-PCR. Our results demonstrated just a single *Leishmania* positive female at sixth day post meal. A total of 1070 female sand flies were exposed in contact with artificial feeder containing the human blood with two different quantities of *Leishmania* parasites: 2.10^6^/mL and 1.10^7^/mL. A blood meal including 1.10^7^/mL LUC-promastigotes was proposed to 270 females and 75 (28%) flies were engorged. Among them, 44 (59%) were positive by RT-PCR analysis, with a relative average of 50551 *Leishmania* parasites. In case of blood feeding of females with 2.10^6^/mL promastigotes, 57 out of 800 (7%) females succeed to feed from artificial feeder which 22 (39%) were positive with a relative average of 6487 parasites.

Leishmaniases are vector-borne diseases caused by obligate protozoan parasites from the genus *Leishmania* (Trypanosomatida: Trypanosomatidae). Leishmaniases are endemic in large areas of the tropics, subtropics and the Mediterranean basin, including more than 98 countries, where there are a total of 350 million people at risk and 12 million cases infected. Canine leishmaniasis is a serious problem and it is estimated that 2.5 million dogs are infected in the Mediterranean basin only (Moreno & Alvar 2002). Among the endemic regions on five continents, there is an estimated incidence of 0.7-1.2 million cases of cutaneous leishmaniasis (CL) and 0.2-0.4 million cases of visceral leishmaniasis (VL) in these countries (Alvar et al. 2012).

Leishmaniasis is transmitted by the bite of infected female sand flies (Diptera: Psychodidae: Phlebotominae) whose hosts are mammals such as canids, rodents, marsupials, hyraxes, or human beings. Among more than 800 sand fly species described, approximately 166 species have been reported to be proven (49 species) or potential (118 spp.) vectors for different pathogenic *Leishmania* parasites in the Old (six *Leishmania* spp.) and New World (14 *Leishmania* spp.) (Akhoundi et al. 2016).

*Leishmania infantum* is the causative agent of infantile visceral leishmaniasis in the Old World *e.g*. Mediterranean region (Marty et al. 2007, Pomares et al. 2016), Middle-East (Hotez et al. 2012), central and south Asia (Strelkova et al. 2015) as well as New World *e.g*. south America (Kuhls et al. 2011) where it has been called *Leishmania chagasi*. It also causes rare cases of CL and mucocutaneous leishmaniasis (MCL) throughout the Mediterranean basin. This etiologic agent of VL has a high prevalence in Europe particularly in the southern regions leading to a latent public health threat (Ready 2010). It has been reported in numerous publications targeting *Leishmania* infection in the symptomatic and asymptomatic dogs (*Canis familiaris*) (Solano-Gallego et al. 2011, Laurenti et al. 2013), rabbit (García et al. 2014), Hare (Molina et al. 2012), etc. as the reservoir. The dogs (*C. familiaris*) are the principal domestic reservoir in southern Europe with an average seroprevalence up to 25% (Maia & Cardoso 2015).

There are numerous sand fly species belonging to genus *Phlebotomus*; subgenera *Larroussius* and *Adlerius* which have been reported as the proven or potential vector of *L. infantum* in the Mediterranean region (Depaquit et al. 2013, Akhoundi et al. 2016). Among them, *Phlebotomus pernicioucus* is one of the known and important proven vectors particularly in south Europe (Prudhomme et al. 2015).

To simulate the leishmaniasis biological cycle in the laboratory conditions, several investigations were conducted explaining the experimental infection of sand fly vectors by *Leishmania* parasites using alive animal hosts (*e.g.* dog, mice, hamster and etc.), and/or artificial feeder (Volf & Volfova 2011, Aslan et al. 2013, Martín-Martín et al. 2015). The most of mentioned studies were concentrated on the cutaneous *Leishmania* species (Belkaid et al. 1998, Mears et al. 2015). Beside these studies, there are some investigations which have been focused on the viscerotropic species (Martín-Martín et al. 2015, Sadlova et al. 2015).

For the sand fly species used for experimental infection, *P. orientalis* (Sadlova et al. 2015), *P. pernicioucus* (Guarga et al. 2000), *P. longipalpis* (Maia et al. 2011) have been selected as the suitable candidates for simulation of Old World leishmaniasis in the laboratory condition.

Some investigations have applied the labelled transfected *Leishmania* species for improving the knowledge on the biology of parasites in sand fly and animal hosts in in vivo and in vitro experiments.

In the present study, we coupled for the first time, an experimental infection of *P. pernioucus* by bioluminescent *L. infantum* using BALB/c mice and artificial feeder in order to monitor experimental infection and to quantify the relative sand fly infection rate analysing by real-time polymerase chain reaction (RT-PCR).

## MATERIALS AND METHODS

*Sand fly colony* - In the present study, we colonised *P. perniciosus* coming from Dr R Molina insectarium (Instituto de Salud Carlos III, Spain). We maintained different biological stages of egg, larvae, pupae and adult under controlled conditions of incubator including 26-27ºC temperature, 10/14 h light/dark photoperiod and > 70% relative humidity.

The freshly emerged adults were transferred by a mouth aspirator in the insect rearing cages (BugDorm-1, 30x30x30cm and 24 x 24 mesh/square inch). Cotton soaked with a sterile sugar solution (30%) was offered them permanently.

The BALB/c mice anesthetised intraperitoneally with ketamine/xylazine (150 mg/kg and 15 mg/kg) were used for female nutrition once per week. Moreover, the glasses artificial feeders were served using 10 mL of human blood purchased freshly from Etablissement Français du Sang (EFS). For simulation of the host skin, the porcine intestinal membrane-previously disinfected by 70% ethanol and sterile physiological saline-were used. In order to prevent any coagulation, the blood was kept at 37ºC using water flowing system. The female sand flies were fed twice per week for one hour. The blood-fed females were transferred from the original cage into a new plastic oviposition pot with an equal number of males for mating and egg laying. The larvae were fed with a mixture of rabbit feces and pellets as previously described (Volf & Volfova 2011).

*Parasites culture* – *L. infantum* strain MON-1 (MHOM/FR/94/LPN101) with transgenic modification-expressing luciferase (LUC-parasites) was routinely maintained and passaged (Michel et al. 2011). *L. infantum* promastigotes were cultivated in incubator at 26ºC in M199 medium supplemented with adenosine 0.1 mM, biotin 1 µg/mL, bovine hemin 5 µg/mL, streptomycin 100 µg/mL, penicillin 100 U/mL, 2 µg/mL biopterin, L-glutamine 2 mM, folic acid 10 µg/mL and 10% fetal calf serum (culture medium) (Michel et al. 2011). Exponential growth phases were anticipated before parasite inoculations into mice (2.10^8^ metacyclic promastigotes) or experimental infection with artificial feeder (2.10^6^ and 1.10^7^ metacyclic promastigotes/mL of human blood).

*Mice, inoculation of LUC-parasites and ethics statement* - Five BALB/c mice with seven weeks old were purchased from Charles River (France) and maintained under specific pathogen-free, dietary and stable climatic conditions. They were kept under surveillance according to the regulations of the European Union, the French Ministry of Agriculture and to Federation of Laboratory Animal Science Associations (FELASA) recommendations. The experiments were approved by the ethics committee of the Nice School of Medicine, France (Protocol number: NCE/2014-189). BALB/c mice were inoculated by 2.10^8^ luciferase-transgenic promastigotes via intravenous route (IV) in the tail base.

*Bioluminescence imaging* - Mice infected with LUC-parasites were imaged using the Photon Imager (Biospace Lab, France). Luciferin solution (300 mg/kg) was injected to mice via intraperitoneal (IP) route in order to permit observing and monitoring the emission of *Leishmania* bioluminescence. Ten minutes after the Luciferin injection, the mice were anesthetised by 5% isoflurane/1L, O_2_. Min^-1^ atmosphere and put then directly in the imaging chamber of the Photon Imager with 2% isoflurane/0.2L O_2_. per mouse min^-1^ atmosphere.

Acquisition of emitted photons radiated by infected mice, with a charge-coupled device camera, were monitored for 20 min in previously defined regions of interest (ROI) that delimited the surface of analysis.

*Sand fly xenodiagnoses with infected mice* - The LUC-parasite infected mice previously confirmed by imaging were selected for sand fly infection.

BALB/c mice with different post-infection dates were anesthetised intraperitoneally with ketamine/xylazine (150 mg/kg and 15 mg/kg) and placed individually for 45 min in the cage directly in contact with 120 to 200 female *P. perniciosus* (four to seven days old) and equal number of males for each test.

The blood-fed females were then separated after 24 h and transferred to oviposition pots containing equal male numbers and maintained in incubator under controlled conditions as mentioned above (Hlavacova et al. 2013).

*Sand fly infections with artificial feeder* - For each feeding, 10 mL human blood containing 2.10^6^/mL or 1.10^7^/mL LUC-metacyclic promastigotes were proposed directly via the cage mesh to 120 to 200 female sand flies during one hour. The blood-engorged females were separated and kept as described above. The dead females were conserved at -20ºC for the further molecular analyses.

*DNA extraction and RT-PCR* - Each blood-fed female sample was put in a sterile tube of Lysing Kits (Precellys®) containing 100 µL sterile water and 20 µL Chelex 5% and then homogenised by Precellys® (2 x 30 sec, with a break of 15 sec).

After centrifuging at 8000 rpm for five min, the whole solution was transferred into a new 1.5 mL microtube and incubated at 90ºC for 40 min. The second centrifugation was carried out at 15000 rpm for 10 min. Then the supernatant of diphasic solution was transferred into a new 1.5 mL microtube and kept at -20ºC.

RT-PCR was implemented for detection and quantification of *L. infantum* targeting minicircle kinetoplast DNA (kDNA). Primers and probe previously described by Mary et al. (2004) comprising 20 pmol of each forward (5’-CTTTTCTGGTCCTCCGGGTAGG-3’) and reverse (5’-CCACCCGGCCCTATTTTACACCAA-3’) primers and 3.33 pmol of TaqMan probe (FAM-TTTTCGCAGAACGCCCCTACCCGC-TAMRA) were used for *Leishmania* screening and quantification. The assays were performed with a final volume of 10 µL including 2.5 µL DNA sample.

The standard curve was obtained from the primary DNA extraction source of 2.5.10^7^ parasites and diluted serially (six times) with 1/10 rate which corresponding to the 50000 to 0.05 parasites in 2.5 µL. RT-PCR program was implemented in two steps temperature of 95ºC and 60ºC for 40 cycles. A pair of positive and negative controls was used for each assay.

## RESULTS

**Fig. 1:**
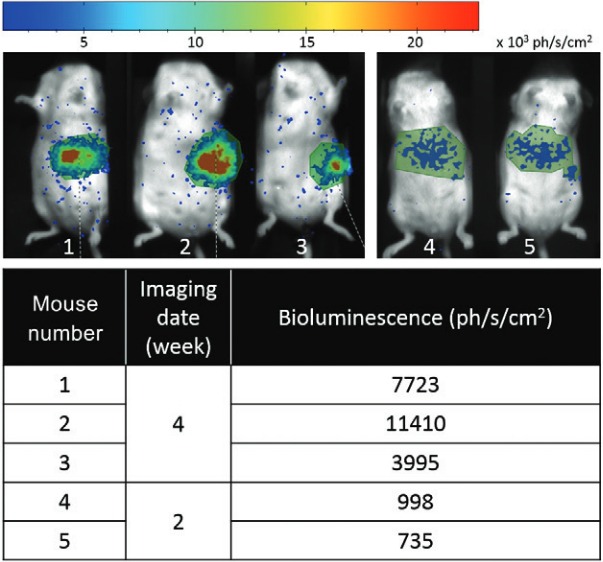
: in vivo monitoring of LUC - *Leishmania infantum* in the region of interest of the mice’ target organs.

*In vivo screening of LUC-L. infantum in infected mice* - BALB/c mice were inoculated by 2.10^8^ LUC-*Leishmania* promastigotes via IV route according to the protocol, described above. The infected mice were imaged and monitored for the presence of the luminescence (expressed as photons/s/cm2) particularly in ROI of the target organs such as the liver or spleen. The mice infection was verified from two to four weeks post IV inoculation (Fig. 1).

*Sand fly xenodiagnoses with infected mice* - Five infected mice were anesthetised and proposed in contact to a total of 1840 new emerged female sand flies at different dates of mice post infection. Among them, 428 females were fed blood meal and after their death, they were individually analysed by RT-PCR. Our results demonstrated just a single female *Leishmania* positive at sixth day after blood meal (Table I)*.*

**TABLE I:** The characteristics of different experimental infections of *Phlebotomus perniciosus* by LUC - *Leishmania infantum*

Experimental model	Promastigote load	Experimentation	Fed sand flies(%)		*Leishmania* positive sand flies/Fed sand flies		Average *Leishmania* number (extremes)	
Artificial feeder	1.10^7^/mL of blood	1	57/150 (38%)	75/270 (28%)	32/57 (56%)	44/75 (59%)	32015 (11-287573)	50551 (11-481978)
		2	18/120 (15%)		12/18 (66%)		99982 (125-481978)	
	2.10^6^/mL of blood	1	10/200 (5%)	57/800 (7%)	4/10 (40%)	22/57 (39%)	134 (10-458)	6487 (10-65825)
		2	12/200 (6%)		4/12 (33%)		129 (18-341)	
		3	20/200 (10%)		9/20 (45%)		6322 (20-65825)	
		4	15/200 (7.5%)		5/15 (33%)		11300 (256-34801)	
Murin model	2.10^8^ (IV route)	Mouse 1 (8 week post infection)	17/120 (14%)	428/ 1840 (23%)	/	1/428 (0.2%)	/	11
		Mouse 1 (10 week p.i)	15/200 (7.5%)		/		/	
		Mouse 1 (11 week p.i)	10/200 (5%)		/		/	
		Mouse 2 (8 week p.i)	58/120 (48%)		/		/	
		Mouse 2 (11 week p.i)	21/200 (10.5%)		/		/	
		Mouse 3 (21 week p.i)	16/200 (8%)		/		/	
		Mouse 3 (30 week p.i)	29/200 (14.5%)		/		/	
		Mouse 4 (2 week p.i)	147/200 (78%)		1/147 (0.7%)		11	
		Mouse 4 (2 week p.i)	67/200 (33.5%)		/		/	
		Mouse 5 (2 week p.i)	48/200 (24%)		/		/	

*Sand fly infection with artificial feeder* - A total of 1070 new emerged female sand flies were exposed in contact with artificial feeder containing the human blood with two different quantities of *Leishmania* parasites: 2.10^6^/mL and 1.10^7^/mL.

A blood meal including 1.10^7^/mL LUC-promastigotes was proposed to 270 females and 75 (28%) flies were engorged. Among them, 44 (59%) were positive by RT-PCR analysis, with a relative average of 50551 *Leishmania* parasites (Table I). The mean of *Leishmana* number in infected *P. perniciosus* at different days of p. i. are presented in the Table II. The maximum mean (130164) of parasites, obtained by fed flies were seen at d8 p.i. (Table II).

**TABLE II:** The average of *Leishmania* number in the infected *Phlebotomus perniciosus* in different days of post infection

Experimental model	Promastigote load	Days post infection	Infected sand fly number	*Leishmania* number/day p.i. (mean/median)
Artificial feeder	1.10^7^/mL of blood	d6	7	28665/3310
		d8	8	130164/36080
		d10	22	17762/2091
		d11	3	62434/1598
		d13	4	101075/49778
	2.10^6^/mL of blood	d6	2	17580/17580
		d8	3	3431/1407
		d10	4	134/35
		d11	13	7440/341

Among 800 female sand flies prospected by 2.10^6^/mL promastigotes, 57 (7%) female flies succeed to feed from the artificial feeder which 22 (39%) were positive with a relative average of 6487 *Leishmania* parasites (Table I). The details of *Leishmania* number at various post infection dates are given in the Table II.

**Fig. 2:**
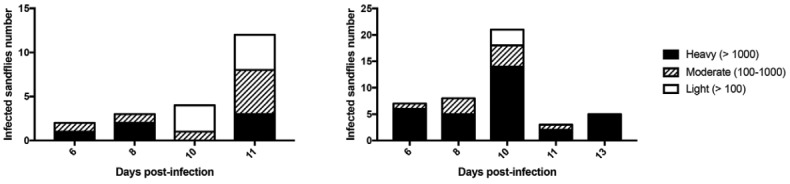
: infection intensity of female *Phlebotomus perniciosus* (light: < 100, moderate: 100-1000, heavy: 1000) in different dates of post infection analysed by a real-time polymerase chain reaction). (A) 2.106/mL LUC-promastigotes; (B) 1.107/mL LUC- promastigotes.

**Fig. 3:**
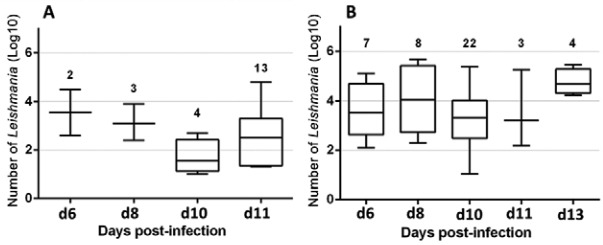
: the *Leishmania infantum* promastigote loads at different days of post infection of female *Phlebotomus perniciosus*, prospected by real-time polymerase chain reaction. (A) 2.106/mL LUC-promastigotes; (B) 1.107/mL LUC-promastigotes. The number of infected flies is indicated in the above of each day.

The post infection intensities of female *P. perniciosus* in different dates are shown in the Figs 2-3. With considering the whole infected females in each condition of 2.10^6^/mL and 1.10^7^/mL, we found 27% and 68% of heavy infections (> 1000 promastigotes) respectively (Figs 2-3).

## DISCUSSION

In vivo imaging techniques are the valuable tools which are used in the recent decade. These methods have been applied for real time screening of labelled invasive microorganisms in living animals. In the recent years, they have been served for in vitro and in vivo monitoring of *Leishmania* spread in the invertebrate sand fly vectors and vertebrate animals (Guevara et al. 2001, Sadlova et al. 2011). For this purpose, *Leishmania* parasites have transfected with the genes of fluorescent proteins such as GFP, RFP and luciferase and used for the studies targeting experimental infection (Kimblin et al. 2008, Sadlova et al. 2011, Calvo-Álvarez 2012, Taheri et al. 2015). These methods have some advantages including the (i) monitoring diseases along the time without sacrificing the living animal, (ii) visualising the *Leishmania* infection in the target organs, (iii) allowing repetitive observation of metabolically active cells due to non-destructive and non-invasive characteristics, (iv) screening in real time the drug efficacy in qualitative assessment of an infection.

In the present study, we used for the first time, luciferase transfected *L. infantum* for experimental infection of *P. pernicious -* as the proven vector - by murine model and artificial feeder. Lang et al. (2005) reported the successful utilisation of *L. amazonensis* recombinants stably expressing the firefly luciferase gene for their experiments including (i) in vitro drug screening on the clinically relevant stage of the parasites (*i.e.* amastigote-loaded mouse bone marrow derived macrophages), and (ii) the monitoring of the parasitic process in living animals. Moreover, they indicated that both control and luciferase-expressing parasites are driving the same clinical processes in BALB/c mice. Hutchens and Luker (2007) in their review paper reported several cases of application of bioluminescence imaging for studying not only on the infectious diseases, but also, on the bacterial infections *e.g. Salmonella typhimurium.* Based on their reports, pathogens, insects and mammalian cells can be engineered to express one or more luciferase enzymes as reporters for in vivo imaging. In the other study conducted in same year, Lecoeur et al. (2007) with drug treatment using aminoglycoside ointment (a topical treatment of CL) against luciferase transgenic *L. major*, explained in details the advantage of this technique as a robust method to rapidly assess efficacy of drugs/compounds, to screen treatment modalities and to allow standardised comparison of different therapeutic agents. After these studies, different engineered viscero - and dermotropic *Leishmania* species to express luciferase were widely used with mice models for screening the *Leishmania* infection, quantifying the parasites, drug therapies (Thalhofer et al. 2010, Talmi-Frank et al. 2012, Reguera et al. 2014, Reimão et al. 2015). Taking advantages of mentioned studies, we used the bioluminescent *L. infantum.* The murine model prospected in the present study was a VL model without cutaneous clinical lesion. Despite 428 females flies fed on the infected mice, we found only one female with a low load relative number of *Leishmania* equivalent to 11 parasites. Sadlova et al. (2015) studied the same model by injecting *L. donovani* parasites derived from *P. orientalis* into the ear of BALB/c mice. According to their analysis, those female sand flies which were fed on the infected (17% *Leishmania* positive from nine-15 weeks post infection) and collateral (positive pool of sand flies) ears were the *Leishmania* positive samples.

Maia et al. (2011) compared the experimental transmission of two strains of dermotropic and viscerotropic *L. infantum* by two vectors of *Lutzomyia longipalpis* and *P. perniciosus.* They used an artificial feeder contained rabbit blood with 1.10^7^/mL promastigotes. They obtained an average of 65768 parasites with viscerotropic strain. We had a similar experimental condition with them *e.g.* the same temperature (26ºC) and relative humidity (70%) and the number of viscerotropic promastigotes/mL blood (1.10^7^/mL). Our findings showed the average of 50551 promastigotes which were close to the results of Maia et al. (2011).

In the other study carried out by Aslan et al. (2013), they suggested infected blood containing 5.10^6^ *L. infantum* /mL to *L. longipalpis.* They found, at 8th days post infection, an average of 2.10^4^, 8.10^4^ and 4.10^4^ parasites in the seven, nine and 13 artificially infected sand flies respectively. Based on our results with a blood meal containing 1.10^7^ parasites/mL, we obtained an average of 30164 parasites at d8 p.i. which is in the range of 2,0000 and 8,0000 parasites reported by Aslan et al. (2013) for the same day.

Based on the results obtained in the present study, there was a wide variability in infection intensity of *P. perniciosus* and their acquired *Leishmania* parasite burden. The artificial feeder seems to be an efficient method to obtain high rate of infectivity. Several factors can play the role in this phenomenon such as the type of *Leishmania* (viscerotropic or dermotropic) and sand fly species, their maintenance condition *e.g.* temperature, the proposed number of parasite detected by RT-PCR and bioluminescence imaging.
